# The Association Study between Twenty One Polymorphisms in Seven Candidate Genes and Coronary Heart Diseases in Chinese Han Population

**DOI:** 10.1371/journal.pone.0066976

**Published:** 2013-06-26

**Authors:** Barrak F. Alobeidy, Cong Li, Alya A. Alzobair, Tao Liu, Junzhang Zhao, Yuan Fang, Fang Zheng

**Affiliations:** 1 Center for Gene Diagnosis, Zhongnan Hospital of Wuhan University, Wuhan, Hubei, P.R. China; 2 Department of Internal Medicine, College of Medicine, Mosul University, Mosul, Iraq; Yale School of Public Health, United States of America

## Abstract

Previous genome-wide association studies (GWAS) in multiple populations identified several genetic loci for coronary heart diseases (CHD). Here we utilized a 2-stage candidate gene association strategy in Chinese Han population to shed light on the putative association between several metabolic-related candidate genes and CHD. At the 1^st^ stage, 190 patients with CHD and 190 controls were genotyped through the MassARRAY platform. At the 2^nd^ stage, a larger sample including 400 patients and 392 controls was genotyped by the High Resolution Melt (HRM) method to confirm or rule out the associations with CHD. MLXIP expression level was quantified by the real time PCR in 65 peripheral blood samples. From the 21 studied single nucleotide polymorphisms (SNPs) of seven candidate genes: *MLXIPL, MLXIP, MLX, ADIPOR1, VDR, SREBF1* and *NR1H3,* only one tag SNP rs4758685 (T→C) was found to be statistically associated with CHD (*P-*value = 0.02, Odds ratio (OR) of 0.83). After adjustment for the age, sex, lipid levels and diabetes, the association remained significant (*P-*value = 0.03). After adjustment for the hypertension, *P-*value became 0.20 although there was a significant difference in the allele distribution between the CHD patients with hypertension and the controls (*P-*value = 0.04, 406 vs 582). In conclusion, among the 21 tested SNPs, we identified a novel association between rs4758685 of *MLXIP* gene and CHD. The C allele of common variant rs4758685 interacted with hypertension, and was found to be protective against CHD in both allelic and genotypic models in Chinese Han population.

## Introduction

In the recent years, more than 700,000 deaths are caused by the consequences (e.g. myocardial infarction) of CHD each year in China, according to the estimation of the World Health Organization. To identify genetic variants that affect the risk of CHD will advance our understanding of how environmental and genetic factors interplay together to lead to CHD. Several genetic loci have been identified for CHD, including SNPs on chromosomes 1p13, 1q41, 2q36, 3q22, 6p24, 6q25, 9p21, 10q11, 12q24 and 15q22, these loci are associated with increased risk of CHD or myocardial infarction based on large-scale GWAS investigations of multiple ethnic populations [1]–[9]. However, most of the findings were identified and confirmed in the European ancestry populations, while a comparatively smaller number of genetic loci associated with CHD have been reported in the Chinese population.

Consequently, we designed the current study in which we aimed to genotype 21 SNPs in seven candidate genes loci: *MLXIPL* (gene ID51085), *MLXIP* (gene ID22877), *MLX* (gene ID6945), *ADIPOR1* (gene ID51094), *VDR* (gene ID7421), *SREBF1* (gene ID6720) and *NR1H3* (gene ID10062) in Chinese Han population. The candidate genes were selected by a pathway-driven approach of reviewing literatures, in which they were reported to be involved in the pathways implicated in the lipid and glucose homeostasis. MLXIPL (MLX interacting protein-like) and MLX can form heterodimers, then activate the transcription of fatty acid synthesis genes in a glucose-dependent manner [10]. A meta-analysis combining 3 GWAS identified an association between common variants near *MLXIPL* gene and the plasma triglyceride concentration conducted with over 8,000 individuals of European ancestry [6]. And a significant association was also found between *MLXIPL* gene polymorphisms and CHD in Chinese Han population [11]. Considering the similar functions of MLXIP and MLXIPL as intracellular metabolic sensors through binding with MLX, we hypothesize that *MLXIP* gene polymorphisms also confer risk to CHD. *ADIPOR1* encoding an adiponectin receptor, is speculated to play a role in the adipocytokine signaling pathway [12]. *VDR* is remarkably highly expressed in the hippocampus and may influence the neural development or activity of feeding. *SREBF1* (sterol regulatory element binding transcription factor 1) and *NR1H3* (liver X nuclear receptor alpha variant 1) are both well-known lipogenic genes [13], [14].

## Materials and Methods

### Subjects

In our study, we carried out a 2-stage association for CHD in the Chinese Han population. The discovery panel at the 1^st^ stage contained 190 patients and 190 unrelated non-CHD individuals, comparatively smaller than the 2^nd^ stage panel (400 CHD patients and 392 controls). All samples were collected from Zhongnan Hospital of Wuhan University and Asia Heart Hospital between January and June of 2011 in Wuhan, China, and they were randomly selected and assigned to discovery and replication panels.

All CHD patients underwent diagnosis of coronary angiography; CHD was defined as ≥50% stenosis in one or more major coronary artery, medical records of percutaneous coronary angioplasty, coronary artery bypass graft or myocardial infarction. Patients with myocardial bridge, congenital heart diseases or other types of atherosclerotic lesions were excluded. Hypertension was defined as a clinical blood pressure of ≥140/90 mmHg or history of medication. Type 2 diabetes was diagnosed by a fasting blood glucose level of ≥7 mmol/L. All study participants are of the ethnic Han origin by self-report. Relevant data were also collected from all the participants by direct interviews or from medical-case files including age, gender, lipid profiles, history of hypertension, type 2 Diabetes and dyslipidemia. Clinical examinations were carried out using rest electrocardiograms (ECG) for the control participants. All control participants showed no signs of CHD, hypertension, diabetes mellitus or dyslipidemia based on the ECG results and medical-case files at the time of enrollment. Blood pressure was measured using a standard mercury sphygmomanometer on the left arm after 5 min rest in the sit position. Fasting blood glucose (FBG), total cholesterol (TC), total triglyceride (TG), high density lipoprotein (HDL)-cholesterol (HDL-C), low density lipoprotein (LDL)-cholesterol (LDC-C), apoliprotein (apo)AI, apoB, lipoprotein (Lp)(a) and C reactive protein (CRP) were analyzed by standard techniques, which were employed by the Core Laboratories in Zhongnan Hospital and Asia Heart Hospital. The study was approved by ethics committee of Zhongnan Hospital of Wuhan University and met the declaration of Helsinki.

### SNP Selections and DNA Extractions

The SNP selections were based on Haploview 4.2 program [15] and Chinese population data from the HapMap database (http://www.hapmap.org, phase2, HapMap-HCB). Totally 21 SNPs, most of which were tag SNPs, were selected for genotyping, tag SNPs were identified in the HapMap HCB data using pairwise r^2^ threshold of ≤0.8 and minor allele frequency (MAF) threshold of ≥0.1. The 21 SNPs located in 7 genes: *MLXIPL* (rs7798357, rs11760752, rs6460045), *MLXIP* (rs4758685, rs4758684, rs10847689, rs7978353), *MLX* (rs665268), *ADIPOR1* (rs2275735, rs10920532, rs1342387, rs4989513, rs16850799), *VDR* (rs1540339, rs2239184), *SREBF1* (rs11868035, rs4925119) and *NR1H3* (rs2279238, rs7120118, 12221497, rs11039149).

Blood samples were drawn from study participants and genomic DNA was isolated via the standard proteinase K digestion and phenol-chloroform extraction.

### Genotyping

At the 1^st^ stage, genotyping was performed in a discovery panel of 190 patients and 190 controls using MassARRAY platform (Sequenom Inc., San Diego, CA, USA). The data analysis including automated allele calling was done using MassARRAY® Typer Genotyping Software (Sequenom, Inc., San Diego, CA, USA). All the samples were tested in duplicate. At the 2^nd^ stage, the HRM method was performed to genotype 400 CHD patients and 392 controls to confirm or rule out the association of the target SNPs, which were revealed to be nominally significantly associated with CHD at the 1^st^ stage. A total of 50 DNA samples (from both cases and controls) were randomly selected for verification of genotyping results using direct PCR sequencing (Life Technologies Corporation, Shanghai, China).

### MLXIP Expression Determination

The total RNA of peripheral leukocytes from 65 healthy individuals was extracted using RNA Purification Kit (Kangwei Century Reagent Company, Wuhan, China). The *MLXIP* expression level was measured by real-time PCR using SYBR_ Green PCR Master Mix (Applied Biosystem, Foster City, CA, USA) and Mx3000p (Agilent Technologies, Inc. Santa Clara, CA, USA). All the samples were assayed in triplicate and *β-actin* was used as an internal control to normalize the results. The 2^−ΔCt^ was used as the comparative expression level by comparative threshold cycle (Ct) method, and ΔCt was the difference in the threshold cycles for *MLXIP* and *β-actin*.

### Statistical Analysis

SNPs genotypes were tested for Hardy-Weinberg equilibrium among patients and controls using χ^2^ test. Allelic and genotypic associations of SNPs with CHD were assessed using Pearson’s 2×2 and 2×3 contingency table χ2 test (SPSS, version 18.0). ORs and 95% confidence intervals (CIs) were estimated using the χ2 test (SPSS, version 18.0). When the case-control samples were stratified, Breslow-Day tests were performed to analyze the homogeneity between ORs from each sub-group (SPSS, version 18.0). Multivariate analysis was performed by incorporating age, sex, hypertension and type 2 diabetes covariates by using multivariate logistic regression (SAS, version 8.1). The expression levels of *MLXIP* gene was compared in different subgroups by Mann-Whitney test. Empirical p-values were determined using the PLINK v1.06 program with 100,000 Monte Carlo simulations.

## Results

### Clinical Characteristics of the Study Population

The clinical characteristics of the combined study cohort were detailed (Table 1). The proportion of male and the average age were both significantly higher in CHD patients than that of controls. Therefore, we applied Breslow–Day homogeneity tests to test modifier effect of gender and age, and both did not show heterogeneity. Significant higher ratios of hypertension, Diabetes and hyperlipidemia were observed in the CHD patients compared to the controls. Since most patients with hypertension have been through antihypertensive therapy, the proportion of hypertensive patients were reported instead of blood pressure values. Plasma concentrations of TC, LDL-C and HDL-C were significantly lower, while TG, apoAI, apoB, Lp(a), CRP and FBG were significantly higher in the case group than those of the control group (Table 1).

**Table 1:** The characteristics of the clinical data in comparisons between control and CHD groups.

Clinical data characteristics*	Controls n = 582	Patients n = 590
**AGE** (years)	56.64±9.39	60.54±9.35
**SEX** (M%)	56	68
**HT** (%)	0	69
**DM** (%)	0	23
**Hyperlipidemia** (%)	0	18
**TC** (mmol/L)	4.40±0.45	4.30±1.06
**TG** (mmol/L)	1.04±0.31	1.61±0.89
**HDL** (mmol/L)	1.30±0.24	1.09±0.34
**LDL** (mmol/L)	2.62±0.31	2.34±0.90
**apoAI** (g/L)	1.37±0.18	1.12±0.24
**apoB** (g/L)	0.74±0.11	0.81±0.25
**Lp(a)** (mg/L)	47.46±81.84	318.44±311.04
**CRP** (mg/L)	0.97±1.66	4.62±9.36
**FBG** (mmol/L)	5.02±0.58	6.12±2.02

* *P*<0.05 for each clinical indicators, by comparing the control group with the CHD group.

### Genotype and Allele Frequencies

No significant deviation from Hardy–Weinberg equilibrium (HWE) was observed for all tested SNPs in the control groups (*P-*value >0.05). SNPs showed nominal significance of *P*<0.05 at the 1^st^ stage were selected as target SNPs for the further validation at the 2^nd^ stage study (Table 2).

**Table 2:** The 1^st^ stage association of 21 SNPs with CHD.

SNP	Locus		Association Signal			Locatedgene
	Chr	Position(Mb)	Allele	MAF	*P* value	
rs665268	17	37.9	A/G	0.31	0.93	*MLX*
rs1342387	1	201.1	A/G	0.34	0.89	*ADIPOR1*
rs2275735	1	201.2	A/G	0.10	0.03*	*ADIPOR1*
rs4989513	1	201.2	C/T	0.43	0.24	*ADIPOR1*
rs10920532	1	201.2	A/G	0.24	0.07	*ADIPOR1*
rs16850799	1	201.2	A/G	0.49	0.61	*ADIPOR1*
rs2239184	12	46.5	C/T	0.32	0.30	*VDR*
rs1540339	12	46.5	A/G	0.27	0.47	*VDR*
rs2279238	11	47.2	A/G	0.43	0.75	*NR1H3*
rs7120118	11	47.2	C/T	0.35	0.57	*NR1H3*
rs11039149	11	47.2	A/G	0.05	0.40	*NR1H3*
rs12221497	11	47.2	A/G	0.08	0.44	*NR1H3*
rs10847689	12	121.2	C/T	0.38	0.45	*MLXIP*
rs7978353	12	121.2	A/G	0.46	0.66	*MLXIP*
rs4758685	12	121.2	C/T	0.39	0.02*	*MLXIP*
rs4758684	12	121.2	C/T	0.18	0.49	*MLXIP*
rs4925119	17	17.7	A/G	0.21	0.76	*SREBF1*
rs11868035	17	17.7	A/G	0.16	0.37	*SREBF1*
rs6460045	7	72.7	A/G	0.01	NA	*MLXIPL*
rs7798357	7	72.7	C/G	0.11	0.91	*MLXIPL*
rs11760752	7	72.7	A/C	0.01	0.17	*MLXIPL*

* *P*<0.05, by comparing the control group with the CHD group. NA: only 2 genotypes were identified in our samples; MAFs were derived from Hapmap-CHB (http://www.hapmap.org, phase2, HapMap-HCB).

At the 1^st^ stage, SNP rs4758685 in *MLXIP* was associated with CHD (*P* = 0.02), and the SNP rs2275735 in *ADIPOR1* was also associated with CHD (*P* = 0.03) (Table 2). At the 2^nd^ stage, significantly higher C allele frequency of rs4758685 in *MLXIP* was found in controls than that of cases (*P* = 0.02) (Table 3).

**Table 3:** Association results for 2 candidate CHD risk loci in stage 1, stage 2 and combined data.

	Number of		rs2275735			rs4758685		
	Cases	Controls	*P* value	OR(CI)	HWE-P	*P* value	OR(CI)	HWE-P
Stage 1	190	190	0.03	0.52(0.30–0.91)	0.88	0.02	0.80(0.62–0.99)	0.30
Stage 2	400	392	0.25	0.68(0.39–0.96)	0.57	0.02	0.87(0.69–0.99)	0.46
Combined	590	582	0.19	0.70(0.35–0.99)	0.69	0.02	0.83(0.71–0.98)	0.73

*P* values and OR (95%CI) were comparing the control group with CHD group under additive model; HWE-P: *P* value of Hardy–Weinberg equilibrium test for controls.

Finally, we combined the 2 stages samples sets, and the combined association data indicated that the CC genotype remained significantly related with the risk of CHD (*P* = 0.02, OR = 0.83, 95% CI of 0.71–0.98) (Table 3). After adjustment for age, gender, history of diabetes mellitus and lipid levels, the adjusted *P* value was 0.03, OR = 0.85 (95%CI of 0.66–0.96); but when the hypertension was included as an adjusted factor, *P* value became 0.20 for the additive model and 0.34 for the recessive model (see Table 4). These results prompted a clue that there might be an interaction between rs4758685 in *MLXIP* and hypertension. Then we compared the allele distributions between the CHD patients with hypertension and controls, in which a significant difference in the allelic distribution between CHD patients with hypertension and controls was found (*P = *0.04, 406 vs 582). For *ADIPOR1* gene SNP rs2275735, there was no significant allelic or genotypic difference between the case and control groups, χ^2^ = 2.66, *P* = 0.10.

**Table 4:** Comparison of three genetic models of inheritance for MLXIP polymorphism rs4758685 associated with CHD.

Model	*P_obs_*	OR (95% CI)	P_adj_*	OR (95% CI)	P_adj_**	OR (95% CI)
Additive	0.02	0.83 (0.71–0.98)	0.03	0.85 (0.66–0.96)	0.20	0.65 (0.33–0.84)
Dominant	0.05	0.79 (0.61–0.93)	0.06	0.71 (0.49–1.02)	0.23	0.71 (0.41–0.89)
Recessive	0.04	0.77 (0.59–0.99)	0.08	0.74 (0.52–1.04)	0.34	0.78 (0.36–0.71)

Abbreviations: obs, observed; adj, adjusted; OR, odds ratio; CI, confidence interval; *P value adjusted for age, sex, Type 2 diabetes and lipid levels; **P value adjusted for age, sex, Type 2 diabetes, lipid levels and Hypertension.

### MLXIP mRNA Expression

**Figure 1:**
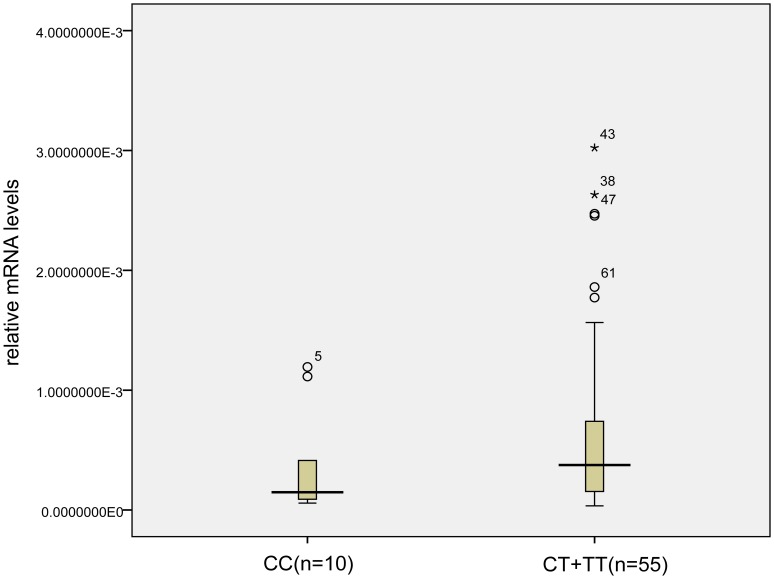
The comparison of MLXIP mRNA expression levels between carriers with CC genotype and CT+TT genotype. The SNP rs4758685 located in the 3′UTR of MLXIP and the mRNA expression level is tend to be lower in the CC genotype carriers than in the CT+TT genotype carriers.

SNP rs4758685 located in the 3′UTR of *MLXIP*, we hypothesized that the SNP might affect the expression levels of the MLXIP mRNA. To determine the relationship between genotypes of SNP rs4758685 and the expression level of MLXIP, we used a real-time PCR analysis to measure the *MLXIP* expression level in leukocytes from 65 individuals with different genotypes. Then we found the mRNA expression levels of *MLXIP* were close between the CT genotype carriers and the TT genotype carriers (median 0.00040 vs 0.00037), so we combined the CT genotype carriers with the TT genotype carriers, the mRNA expression level of MLXIP was tend to be lower in the CC genotype carriers compared with carriers of the CT or TT genotypes, but the difference was not significant (median 0.00015 vs 0.00038, Z = −1.425, *P* = 0.15) (Figure 1).

### Association between the Biochemical Tests and rs4758685

In order to investigate the functional role of *MLXIP* gene SNP rs4758685, we compared the plasma concentrations of TC, TG, HDL-C, LDL-C, apoAI, apoB, Lp (a), CRP and FBG among the carriers of CC, CT and TT genotypes in the controls. For the cases, we compared the frequencies of CC, CT and TT genotypes carriers between subgroups with normal and abnormal concentrations, since some of the patients took lipid-lowering medicines. No significant associations were observed with *P*<0.05 (data not shown).

## Discussion

In the present study, we examined the association of 21 SNPs, located in 7 genes (*MLXIPL, MLXIP, MLX, ADIPOR1, VDR, SREBF1, NR1H3*) with CHD in Chinese Han population. Among the 21 SNPs, one SNP rs4758685 located in *MLXIP* gene was found to be significantly associated with the risk of CHD. The protective role of rs4758685 C allele against the risk of CHD suggested *MLXIP* as a possible candidate gene involved in the pathogenesis of CHD. Furthermore, the association between MLXIP SNP and CHD was still significant after performing statistical adjustments (for age, sex, Diabetes and lipid levels). However, when we included hypertension as an adjusting factor, the association between SNP rs4758685 and CHD became not significant, instead we got a significant difference in distributions of the SNP between controls and the hypertensive CHD patients. These results suggested that rs4758685 might contribute to CHD through blood pressure alteration.

Till the moment, little information is known about the role of *MLXIP* gene in the diverse physiological or pathological processes. It was reported that *MLXIP* gene played a role in the direct control of glycolysis, and *MLXIP* gene controlled the cell growth through regulation of TXNIP [16]–[18]. *TXNIP* expresses in various cells, including endothelial cells, vascular smooth muscle cells (SMCs) and macrophages. *TXNIP* can control the redox status by binding to and inhibiting thioredoxin which is an antioxidant factor, then modulates the function of the apoptosis signal-regulating kinase 1 (ASK1), making cells prone to the oxidative stress of ROS (reactive oxygen species) [19]–[21]. Recently Ferreira et al. reported that there was an association between TXNIP variations and hypertension in Brazilian general population, and the variations were related to the gene expression in vascular SMCs [22].

We speculated that the SNP rs4758685 might be associated with CHD by interacting with hypertension through the TXNIP pathway. However, how did the SNP rs4758682 exert effects through the TXNIP pathway? We found that the SNP rs4758685 is located in the 3′untranslated region (3′UTR) of *MLXIP* gene, which may affect the stability, localization and translational efficiency of mRNA by providing binding sites for miRNAs [23]. Then the expression level of *MLXIP* was analyzed in our study and we found a tendency of lower expression in the subgroups of CC genotype carriers. It was interesting that the MLXIP expression levels were similar in the CT and TT genotype carriers, and comparatively lower in the CC genotype carriers, though the difference was not significant. Therefore, we hypothesized that SNP rs4758685 affect the function of *MLXIP* gene by affecting its mRNA expression, finally lead to the oxidative status change through TXNIP pathway. But unfortunately, we could not find any miRNA that might bind to the site of this SNP in the currently available database (http://www.targetscan.org).

Population structures are generally unique in different populations, different modifier genes and gene×environment interactions may lead to population structures. CAD is a complex disease, the effect of rs4758685 on CAD may be exposed or masked by different lifestyles or environmental factors among our study cohorts. In our study, we didn’t find the significant association between the other SNPs and the incidence of CHD among Chinese people. It could be resulted partly from the relatively small sample size in 1^st^ study panel or due to their non functional locations (intronic regions). In other studies, polymorphisms in *MLXIPL*, *SREBF1* and *VDR* gene were reported to be associated with the risk of developing coronary vascular diseases [24]–[27]. However, the SNPs in these studies were different from the SNPs involved in our study.

In conclusion, among the 21 tested SNPs, we identified a novel association between rs4758685 of *MLXIP* gene and CHD. The C allele of common variant rs4758685 was found to be protective against CHD in both allelic and genotypic models in the Chinese Han population. Furthermore, we have observed an interaction between rs4758685 and hypertension. We recommend further analysis of the SNP, with a larger sample and multi-center trial, in order to confirm the association with the incidence of CHD and reveal the mechanisms of the association.
